# Local co-ordination and case management can enhance Indigenous eye care – a qualitative study

**DOI:** 10.1186/1472-6963-13-255

**Published:** 2013-07-03

**Authors:** Mitchell D Anjou, Andrea I Boudville, Hugh R Taylor

**Affiliations:** 1 Indigenous Eye Health Unit, Melbourne School of Population Health, The University of Melbourne, Level 5, 207 Bouverie Street, Carlton, Melbourne, VIC 3010, Australia

**Keywords:** Indigenous Australians, Aboriginal and Torres Strait Islander, Eye care, Co-ordination, Case management

## Abstract

**Background:**

Indigenous adults suffer six times more blindness than other Australians but 94% of this vision loss is unnecessary being preventable or treatable. We have explored the barriers and solutions to improve Indigenous eye health and proposed significant system changes required to close the gap for Indigenous eye health. This paper aims to identify the local co-ordination and case management requirements necessary to improve eye care for Indigenous Australians.

**Methods:**

A qualitative study, using semi-structured interviews, focus groups, stakeholder workshops and meetings was conducted in community, private practice, hospital, non-government organisation and government settings. Data were collected at 21 sites across Australia. Semi-structured interviews were conducted with 289 people working in Indigenous health and eye care; focus group discussions with 81 community members; stakeholder workshops involving 86 individuals; and separate meetings with 75 people. 531 people participated in the consultations. Barriers and issues were identified through thematic analysis and policy solutions developed through iterative consultation.

**Results:**

Poorly co-ordinated eye care services for Indigenous Australians are inefficient and costly and result in poorer outcomes for patients, communities and health care providers. Services are more effective where there is good co-ordination of services and case management of patients along the pathway of care. The establishment of clear pathways of care, development local and regional partnerships to manage services and service providers and the application of sufficient workforce with clear roles and responsibilities have the potential to achieve important improvements in eye care.

**Conclusions:**

Co-ordination is a key to close the gap in eye care for Indigenous Australians. Properly co-ordinated care and support along the patient pathway through case management will save money by preventing dropout of patients who haven’t received treatment and a successfully functioning system will encourage more people to enter for care.

## Background

The path to care is different for each of the main eye care conditions causing vision loss for Indigenous Australians – cataract, refractive error, diabetes and trachoma – and the availability of practitioners, services and settings supporting eye care are known to vary considerably and geographically across Australia [1-6]. There is attrition of patients along the pathways in Indigenous eye care [6,7] and patients are often not able to successfully negotiate a given care pathway [3,8,9]. Higher risk patients are also known to have a greater chance of not successfully navigating care pathways and suffer a greater consequence for failing to do so [8,9].

The 2008 National Indigenous Eye Health Survey (NIEHS) established that Indigenous adults suffer six times more blindness than other Australians but 94% of this vision loss is preventable of treatable and 35% of Indigenous adults have never had an eye examination [1]. Co-ordination has been identified as a key component often missing in the service system [10-12] and patient outcomes can be improved if care is co-ordinated to assist the patient’s journey [12-14]. Co-ordination generally involves the arrangement of and communication between various components in the health system including the service practitioners and facilities [12,15]. Case-management, in addition, operates from the patient perspective to support the patient along their care pathway [16].

The importance of co-ordination in health care settings with Indigenous Australians has been demonstrated in cancer care, mental health, alcohol and drug use problems, lung health, medication management and heart disease [17-21]. The use of case management or care co-ordinators has also been shown to improve health care outcomes for Indigenous Australians [22-24].

There have been repeated calls for improved co-ordination of Indigenous eye health but a sustainable and satisfactory solution has not yet been achieved [25-31]. Since the late 1990s, Regional Eye Health Co-ordinator (REHC) positions have been based in Aboriginal Health Services (AHS) to support the regional co-ordination of eye care [26,27]. Currently less than half the originally established 34 regions have a REHC and many of these staff now only work part-time with a small proportion and insufficient time allocated to eye care. Many deficiencies and inconsistencies in the role and function of REHC have been documented [28,29] and this has resulted in many gaps in the various pathways of eye care [10,11].

This study aims to identify the barriers for effective organisation of eye care and patient support at a local area level and propose sector-supported solutions to improve the co-ordination of eye care in Australia for Aboriginal and Torres Strait Islander peoples.

## Methods

Ethical approval for the project was provided by The University of Melbourne and subsequently by eight ethics committees across Australia [4]. Agreement to conduct the project was provided by the National Aboriginal Community Controlled Health Organisation (NACCHO) and five state and one territory affiliate organization [4,32]. The investigation was conducted in accordance with the Declaration of Helsinki 1975 and the National Health and Medical Research Council Guidelines for Ethical Conduct in Aboriginal and Torres Strait Islander Health Research 2003 [33].

Data was collected through semi-structured interviews and focus groups. Focus groups were conducted at 7 sites in Victoria, 3 urban and 4 rural, and involved 81 community members. Semi-structured interviews were conducted at 21 sites across Australia (including the 7 Victorian sites) and included five states and one territory in urban (n = 6), regional (n = 7) and remote (n = 8) locations. Sites for field work were selected in consultation with NACCHO state and territory affiliates and a deductive process using previous reports to identify existing eye care programs. Twenty nine Indigenous health organisations participated in the project. A total of 289 people working in Indigenous health, eye care, hospital, non-government organisations and government were interviewed. These included AHS staff (n = 98), community health staff (n = 14), optometrists (n = 31), ophthalmologists (n = 25), hospital staff (n = 35), Division of General Practice staff (n = 10), non-government organisation staff (n = 16), NACCHO affiliate staff (n = 12) and government staff (n = 29). Consultations were conducted with REHC who have responsibility to co-ordinate Indigenous eye health and optometrists and ophthalmologists providing visiting services to AHS or receiving support for Indigenous specific eye care.

Semi-structured interview questions and focus group discussion topics were pre-scripted [4] although interviews and discussions were tailored to the participants’ areas of operation, knowledge, experience and interest. Open-ended questions explored barriers and solutions to improve the management, planning and operation of eye care services. The semi-structured interview questions investigated issues about the delivery and provision of eye health services, pathways of care and the co-ordination of visiting and specialist services. Focus group discussion topics related to barriers that impact on participant’s access to eye health services and explored suggestions to improve access to current eye care services.

Data were collated and content analysed using qualitative analysis methods and NVivo software. The thematic analysis also included observations, suggestions and successful examples from the field. Broad themes were identified by the research team as a basis to build specific policy recommendations. These areas were workforce, co-ordination, utilization, primary health care/primary eye care, monitoring and evaluation, governance and social marketing/awareness. Pathway diagrams were established and population needs estimates calculated to support and inform sector discussion around policy considerations.

Policy recommendations were developed subsequently through stakeholder workshops. Three workshops were conducted in the course of the project and 86 individual stakeholders attended one or more workshop. An iterative process was undertaken to refine policy recommendations over a six month period using stakeholder comment and feedback and subsequent circulation of draft ideas to stakeholders. Additionally, 32 stakeholder organisations and federal and state ministers and bureaucrats from eight jurisdictions were further engaged through 38 face-to-face meetings with more than 75 people to elicit final feedback on the project proposals.

The draft recommendations were then widely circulated through Aboriginal health and eye health sectors and to government departments for comment and feedback and this additional input allowed further refinement of the recommendations. The recommendations were published in The Roadmap to Close the Gap for Vision Full Report [4] in January 2012 and disseminated to all the stakeholder organisations that had participated in consultations.

## Results

Table 1 summarises the themes, barriers and solutions identified in this study for improving the co-ordination of Aboriginal and Torres Strait Islander peoples’ eye care.

**Table 1:** Themes, barriers and solutions for improving co-ordination of Aboriginal and Torres Strait Islander peoples’ eye care

**Themes**	**Barriers**	**Solutions**
Pathways of care	Service system complexity	Establish local referral pathways and service directories
	– multiple people	
	– multiple locations	
	– multiple visits	
	Knowledge of pathways	Ensure local referral pathways are known to all service providers
Co-ordination workforce	Wide range of tasks	Sufficient people in each area are appropriately designated, trained and funded to organise services and co-ordinate patients
	Inadequate resources	Sufficient workforce and funding are available to meet population needs
	Inconsistency of roles	Ensure each local area identifies personnel and positions required for proper co-ordination and organisation
Case management	Designation of responsibility	Establish case co-ordination strategy within each Aboriginal Health Service for all patients at high need or referred for surgery
Local eye care co-ordination	Fragmented system elements	Establish mechanisms for co-ordination within local population health structures
	Informal organisational arrangements	Local co-ordination is built on partnerships and agreements with local providers and visiting eye services
	Community engagement	Eye care services are developed and delivered with the engagement of the local community

### Pathways of care

Our field studies illustrated that the provision of eye care involves multiple people and multiple locations. These include the patient and their family and carers, Aboriginal Health Workers (AHW), optometrists, ophthalmologists, hospital staff, clinic staff in AHS, private clinics and public hospitals.

**Figure 1:**
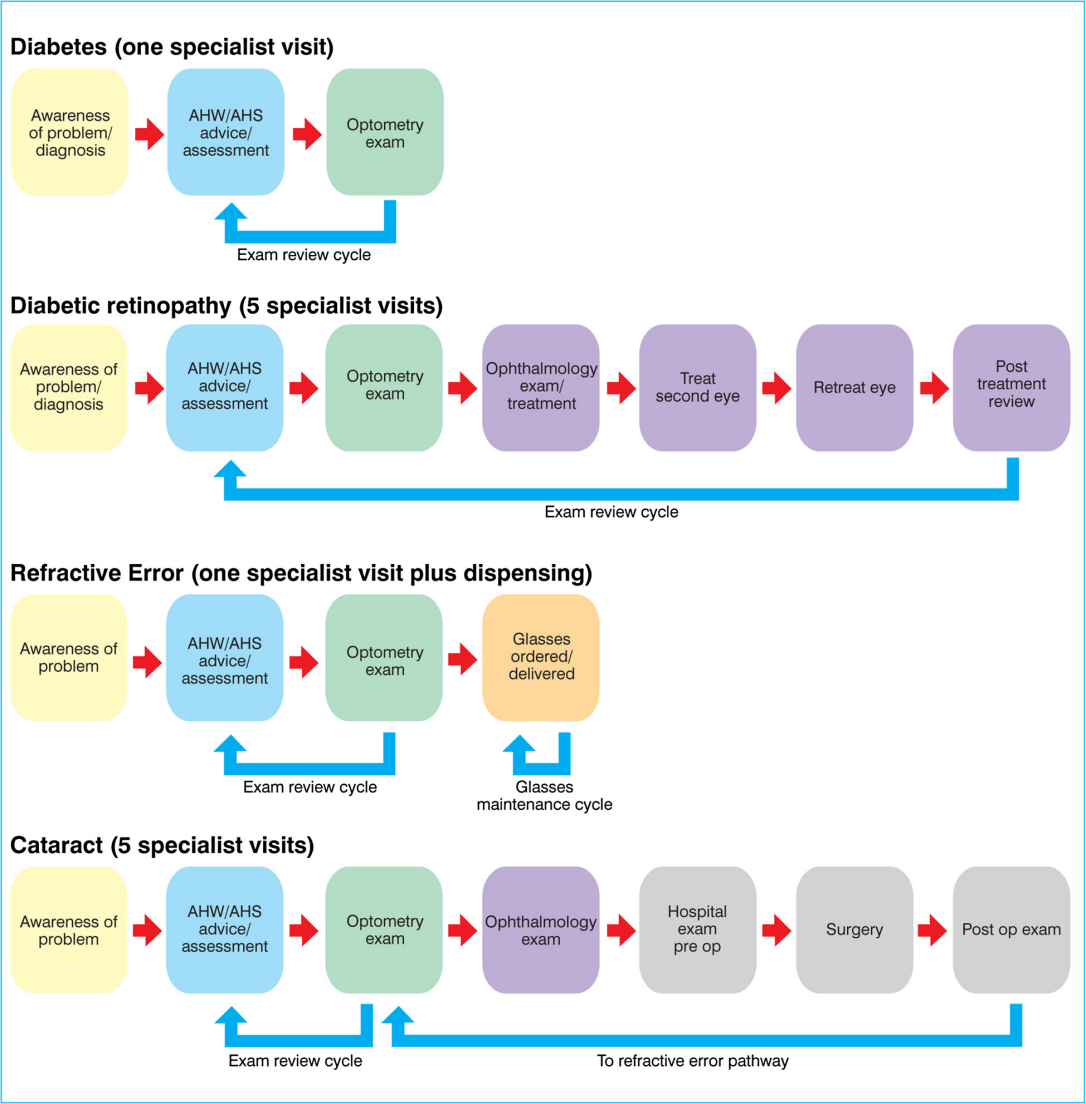
**Complexity of the clinical pathway [**[1]**,**[2]**].**

Multiple visits are usually required to ensure completion of treatment (Figure 1). For example, the ‘normal’ passage of care for someone with cataract may involve six or more specialist visits. It is more difficult to specify the patient pathway in diabetic retinopathy because of the variability of treatment for this condition (one or both eyes treated, pan retinal treatment versus focal macular treatment, limited extent of laser treatment able to be provided at one visit and the need for retreatment). One specialist practitioner working with a largely Indigenous population reported that 46% of patients with diabetic retinopathy required one laser treatment, 29% required two treatments and 24% required three or more treatments.

It was reported by AHS staff that the more steps there are in the care pathway, the greater the likelihood of a patient not successfully traversing the pathway. Each additional provider and location adds complexity and difficulty. It was apparent that poorer outcomes are experienced when the pathway of care is not well understood and therefore not as well supported and cannot be explained readily to patients.

**Figure 2:**
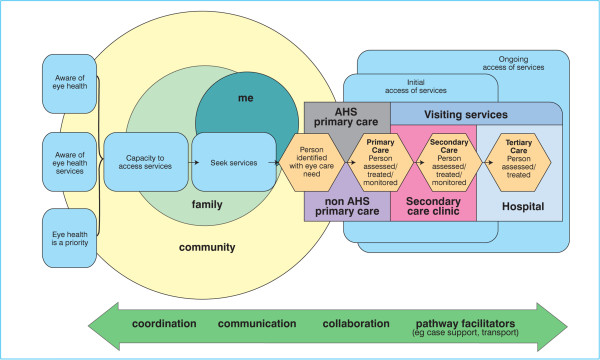
**Patient care pathway [**[1]**,**[2]**].**

A patient care pathway diagram was developed from field consultations (Figure 2). The circular sections to the left represent the interaction between the individual (me), the family and the community and demonstrate the interactions for a person before they enter the service system. The boxes overlying the circles influence whether an individual enters the eye care service system. The service system itself is represented by the overlying boxes to the right, which have a mix of location, level of care, service provider and cycle of care. The relationships between these elements are complex. The arrow at the bottom of the diagram illustrates the mechanisms and behaviours that support the patient journey through the service system – co-ordination, communication and collaboration are key contributors to successful patient care across the system and the role of case management is identified.

Many clinic staff, and referring practitioners, were not aware of the number and type of visits required in care nor the best practice guidelines for a normal or average patient journey. Health staff were aware of their individual responsibilities within a given section of the pathway and their role in identifying, assisting and, if necessary, referring a patient – but they were not well informed about how the other elements of the system worked. Moreover, participants appeared not to be motivated to explore or seek further understanding. This was possibly because of the ever-changing nature of services, but also because of reluctance to step into what was considered another practitioner’s area.

*‘I didn’t know that the visiting optometrist could check eyes for diabetes’* Aboriginal Health Service staff member

The poor understanding of the elements in the system contributes to poorer patient outcomes, variable work quality and a reduced ability for those in the system to advocate for improvement or change. If no-one knows how long it is reasonable to wait for cataract surgery in the local public hospital, then the community just accepts whatever time is offered and the health services and optometrists are not empowered to remonstrate for change.

*‘I have done my job by arranging referral for the patient, it is now not my problem’* Optometrist

Further, the eye care system is made more complex with the mix of public providers and support systems, and private practitioners. Patients are often required to navigate between private and public options for care and sometimes are given information by practitioners who are potentially conflicted. Patients may go to a publicly funded optometry service delivered by a local private practitioner and be referred to a local private ophthalmologist who is required to see the patient before the patient can be placed on a public hospital waiting list for surgery. Again, post-operative follow up may be provided within the private sector. It was observed that the tension between public and private offerings contributes to community distrust of the service system.

*‘We can’t wait for the visiting service, so we go to the local optometrist but we know they are too expensive’* Community member

Recommendations suggested and developed by stakeholders to help better clarify the pathways of eye care included local development of service directories and referral protocols.

### Co-ordination workforce

A wide range of co-ordination and organisation tasks have been identified within regions to ensure effective eye care delivery (Table 2) [4,10]. The specific allocation of responsibility appears to vary from region to region depending on local factors. The number of people required for this co-ordination also varied with local population size, geographic distribution, availability of local services and the requirement for travel. There was consensus demonstrated in our field consultations and stakeholder workshops that the task list and range of responsibilities in co-ordination was generally beyond the capacity and skill set of any one person.

**Table 2:** **The levels of co-ordination to support both eye care services and the patient journey [**[4]**,**[34]**]**

**Levels of co-ordination**	
**Community**	
•	Community liaison provides a vital link between individual community members, their families and the clinic and its services
•	This may include identification, transport, interpretation, translation and moral support
**Clinic, Primary Eye Care**	
•	Referral of more complex cases to visiting eye team
•	Maintenance of patient records and referral lists for visiting eye team
•	Scheduling of visits by visiting eye team
•	Co-ordination with other visiting specialists
•	Co-ordination of exam rooms, accommodation, equipment and local staff
•	Make arrangements for referrals to Regional Hospital
•	Schedule follow up visits as required
**Eye Team, Secondary Eye Care**	
•	Co-ordination of visits with clinic and community
•	Update patient records as necessary
•	Communication and co-ordination between visiting optometrists and ophthalmologists
•	Mechanism for communication and co-ordination with other visiting specialists
•	Specific equipment items brought with team (e.g. lasers, slit lamp)
•	Organise a list/information about patients waiting to be seen
•	Assistance with patient identification, transport, translation, explanation and support
•	Clerical support for forms and paperwork
•	Referral systems for further management and surgery
**Regional Hospital, Tertiary Eye Care**	
•	Organisation of the clinic space, theatre time, staff, accommodation, travel and surgical supplies for the visiting eye teams
•	Co-ordination with other visiting specialists
•	Organisation and supply of surgical equipment
•	Co-ordination of patients who require surgery with community and clinic
•	Organisation of travel and other arrangements for patients
**National/State/Territory**	
•	Co-ordination of other specialist and allied health visits with the visiting team
•	Oversight of co-ordination performed at different levels, recruitment, training and support
•	Oversight of distribution of visiting eye teams (and other specialists) including ratio of optometric and ophthalmic visits and frequency of visits

Using the NIEHS prevalence of diabetes, refractive error, cataract and trachoma in Indigenous Australia, the eye care required each year [1-3] for a cohort of 10,000 Indigenous people has been calculated (Table 3) [11-13]. For clinical service time only 1.0 equivalent full time (EFT) optometrist and 0.3 (EFT) ophthalmologists are required. Travel time and clinical complexity are not included in these estimates. However, some 8.3 EFT staff members are required to provide co-ordination and case management. Site interviews established that many different people are involved and most were not dedicated to eye care. They included community liaison staff, drivers, clinic and hospital booking clerks, nurses and clinic managers. Many of these support staff are already appointed in multifunctional roles but from time to time are needed to assist with eye care; for example, the receptionists and drivers helping when the eye team visits an AHS.

**Table 3:** **Delivery and co-ordination of eye care services for a region with 10,000 Indigenous people [**[4]**,**[34]**]**

		
**Optometry**		
Number requiring glasses examination	640	
Number requiring diabetes eye examination	962	
Number of other eye examinations	98	
**Total Optometry examinations**	**1700**	**1.0 EFT**
**Ophthalmology**		
Number requiring diabetic laser	112	
Number of cataract surgeries	95	
Number of trichiasis surgeries (not in all regions)	36	
**Total Ophthalmology referrals**	**243**	**0.3 EFT**
**Co-ordination**		
Patient liaison (appointments etc.)	3.7	
Patient transport	1.8	
Organising eye clinics	1.3	
Organising hospital	0.1	
Eye clinic support (excludes surgery)	1.5	**8.3 EFT**

Abbreviation: *EFT* Equivalent Full Time.

Regional modelling indicates the number of support people needed but does not specify who should perform the various tasks. It is suggested that the allocation of different responsibilities is determined at the local or regional level taking into account the resources available and the current work and referral patterns.

*‘I am on the road usually 3 out of every 4 weeks…I just can’t get around to everyone…I have a family… and a life’* Aboriginal Health Service staff member (REHC)

Our field interviews in 2010/2011 confirm REHC across the country operate very differently, serve different parts of the eye care service system and that there is not a clear and accepted job description for a REHC [11,27,29]. The work of REHC in regional eye care co-ordination is broadly regarded as invaluable.

*‘As the REHC come, we will leave the eyes to them’* Aboriginal Health Service staff member

In many regions, there are reports of insufficient co-ordination, administration and primary health workers to support the visiting specialist services.

### Case management

Case management was suggested by community members and those working in the service system as a potential solution to the system complexity, operational barriers and difficulty community members have in accessing eye care services. When there was no capacity or interest in supporting community members along the pathway of care, many people failed to get the treatment they required. Case management already exists in AHS for patients with complex needs, such as diabetes. Where case management was provided, it was reported that better patient outcomes were achieved, even with complex and difficult problems.

There was strong sector support for the application of case management resources for eye care directed to high risk patient groups such as those with diabetes and those requiring cataract surgery.

### Local eye care co-ordination

Just as primary care staff were unaware of the referral pathways, optometrists and ophthalmologists, often had little knowledge of the other elements of the eye care system or pathways. These operational silos may be understandable given that optometrists and ophthalmologists generally operate as independent private businesses and may be providing visiting services, but the lack of understanding and linkage was considered detrimental for eye care outcomes.

Where primary health care services were integrated with and supported by specialist services, care was effectively delivered in a timely way. Poorly co-ordinated and organised services tended to discourage patients from seeking and using services.

Currently, there are no links between ophthalmologists and optometrists contracted through the Australian Government’s Medical Specialists Outreach Assistance Program (MSOAP) and Visiting Optometrists Scheme (VOS) who are funded to work in the same geographic area. A lack of shared information and poor communication between specialist eye care providers perpetuated siloed operations, and created barriers between visiting and local services leading to unnecessary duplication.

Good co-ordination between MSOAP ophthalmology services and VOS services has potential to increase efficiency and was supported as a necessary service planning step. Stakeholders agreed also that the selection of priority locations for VOS and MSOAP need to follow a needs-based analysis and have a consistent process for annual review and evaluation.

*‘The eye team comes with the visiting ophthalmologist and then the next week the optometry services are scheduled…it is not well co-ordinated’* Aboriginal Health Service staff member

**Figure 3:**
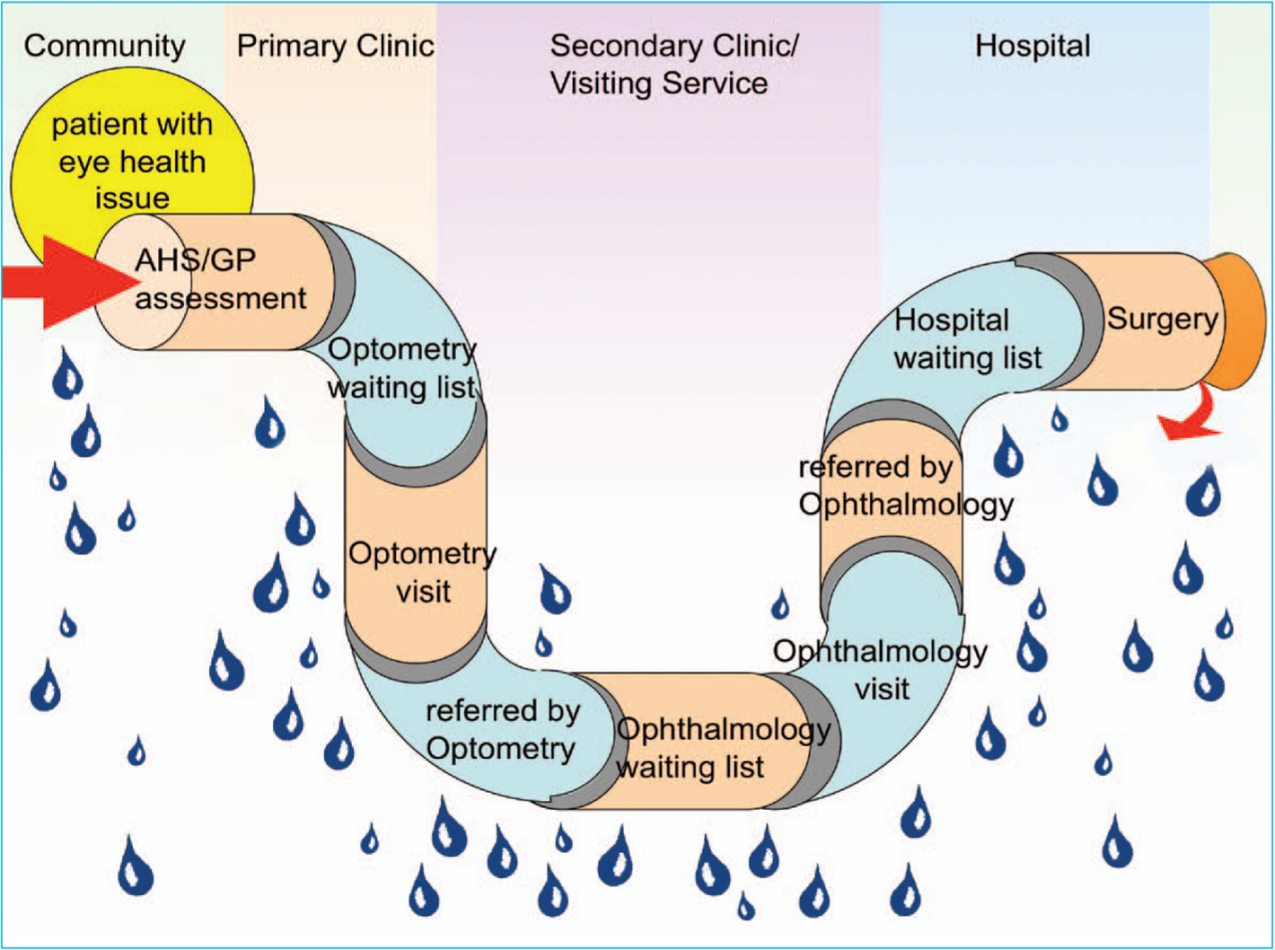
**The ‘leaky pipe’ in the patient pathway for eye care [**[1]**,**[2]**].**

A useful tool to illustrate the difficulties for a patient to successfully traverse the eye care service system and exit with delivery of a treatment outcome at the other end was a leaky pipe (Figure 3) [4,34]. The leaky pipe diagram shows the many steps, providers and locations for an eye care patient and the potential of drop out from the system or ‘leakage from the pipe’. The eye care system is inefficient as patients progress along the system get so far but no further. The cost for the optometry service may be incurred, but the patient does not receive glasses and so is no better off. There are many costs involved in referral for cataract surgery, but if no surgery is performed because the patient drops out of the system, it is very inefficient and wasteful. The diagram also points to the solutions for eye care delivery – the elements of the system need to work closely together and fit into each subsequent element to prevent the leakage and the ‘stopper’, illustrating the lack of cataract surgery services, must be removed to stop the impediment to patient flow along the pipe.

Informal co-ordination arrangements between local providers varied greatly across the country as did expectations of the eye care system. Long waiting times and lists, high fees, and significant travel time and costs were tolerated in some locations in order to receive services, but they would not have been tolerated in other areas. Users in the system were sometimes unaware that they were tolerating an unacceptable service because they were grateful for whatever care they received and knew no different.

Although the practitioners would claim that they did work co-operatively on patient care, we did not observe models with clear leadership of the eye care system at either the local or regional levels. There was also little evidence of eye care teams effectively working together to provide regional care.

A further weakness observed was the dependence on specific individuals and the risk that the service will fall apart when this key individual is absent or leaves.

There was general support in our consultations for formal arrangements between the service elements that would include agreements with clear understandings between providers about the expectations of service response and outcomes.

## Discussion

Poorly co-ordinated health services are inefficient and costly and result in poorer outcomes for patients, communities and health care providers [12,13,35]. Co-ordinated eye care for Indigenous Australians can be achieved through the establishment of clear and shared pathways of care, the provision of sufficient workforce with well identified roles and responsibilities, case management for high needs patients and regional and local management and partnership of services and service providers.

The recommendations to improve the local co-ordination of eye care should be viewed in the broader context of The Roadmap to Close the Gap for Vision which includes 42 linked recommendations to address the full spectrum of Indigenous eye care needs [4]. The Roadmap requires additional annual capped funding of $19.5 million or $68.25 million over five years with staged implementation, two thirds of which is for co-ordination. The provision of adequate co-ordination and an effective governance structure are anticipated to yield tremendous increases in efficiency and dramatically improve patient outcomes. With only a doubling of funding, it is estimated that cataract surgery will increase seven times, diabetic examinations five times and glasses use 2.5 times [4].

The Aboriginal health and eye care sectors supports the development of clear pathways of care so that co-ordination is based on locally established service directories and referral protocols. To be effective these pathways must be made known to all service providers and be consistent with nationally established benchmarks [4].

There needs to be sufficient people in each local area who are appropriately designated, trained and funded to organise and co-ordinate the patient care. Local areas will need to identify the existing or additional personnel and resources required. Reports prepared by Vision 2020 Australia [29] and commissioned by the Office of Aboriginal and Torres Strait Islander Health [28] have proposed better support for the current REHC. However, more people and a greater breadth of responsibilities are required. Additional eye specialists are also required, although the actual increase in the number of optometric and ophthalmic services required for Indigenous eye care is quite small [7]. With the appropriate co-ordination and resources, many specialists would be willing to take on this work [4].

This study identifies that a case management strategy should be considered within AHS for all patients at high need for eye care, those with diabetes and those requiring eye surgery. For patients with diabetes, the case management could be provided by the existing chronic disease co-ordinators. Case management is also required for patients needing cataract surgery as there still remains the large disparity between Indigenous and non-Indigenous people waiting for cataract surgery in public hospitals [36]. The Indigenous Chronic Disease package has established several pilot projects to support case co-ordination in chronic disease [37]. They should specifically include eye care.

The Australian National Health Reforms provide a new platform to initiate mechanisms for the local co-ordination of eye care [38]. Local Hospital Networks and Medicare Locals in consultation with the local AHS can support partnerships and agreements with local service providers and visiting eye services.

The Australian Government has released guidelines for Personally Controlled eHealth Records and one of the aims is to provide better co-ordination of health care across multiple service providers and organisations [39]. Eye health indicators need to be included in these records and act as reminders of the need for eye examinations in high-risk individuals, especially those who have diabetes.

The recommendations we are making are generally consistent with the policy recommendations made previously [26,27] that all too often have not been implemented [25]. However, we have gone into further detail and been even more specific about many of the linkage and co-ordination activities that are required to make the system work. The co-ordination of specialist eye services may act as a template for other areas of health care and the lessons learned could help other specialist services better link with primary care.

The strengths of this study include the broad consensus achieved by regular and detailed consultation with stakeholders, ranging from national organisations and government to individual clients. Over 530 people have contributed to this work. The study was nationwide, covered cities, regional and remote areas and involved semi-structured interviews which allowed participants to proffer solutions for identified problems. The study builds on previous work that has described the value of co-ordination and case management to improve health outcomes [9,11,12,15]. The study was limited by those not included in interview and consultation. The semi-structured interview process provided only limited quantitative information. Many of the sites selected for consultation had successful and existing eye care programs, and, as we were seeking solutions, information and advice from areas without programs was less extensive.

## Conclusions

A significant increase of service provision and utilisation is required to provide eye care services for Aboriginal and Torres Strait Islander peoples at a level equivalent to the Australian population. At present much money is spent in providing services that may not result in any patient benefits; such as an optometric visit but the patient does not receive the glasses they need; a patient referred for cataract surgery they never receive. Properly co-ordinated care and case management can stop this inefficient wastage such that patients will get the care and attention they require when they need it. A good and successfully functioning system will also attract and encourage more people to enter for care. Implementing these changes to improve the co-ordination of eye care is necessary to close the gap for vision.

## Competing interests

The authors declare that they have no competing interests.

## Authors’ contributions

HRT conceived and designed the study. MDA, AIB and HRT participated in developing the study design, conducting the consultations, focus groups and stakeholder workshops and meetings, performed the thematic analysis and drafted the study report. MDA drafted this manuscript. All authors contributed to, read and approved the final manuscript.

## Pre-publication history

The pre-publication history for this paper can be accessed here:

http://www.biomedcentral.com/1472-6963/13/255/prepub
